# Metabolic detoxification and *ace-1* target site mutations associated with acetamiprid resistance in *Aedes aegypti* L

**DOI:** 10.3389/fphys.2022.988907

**Published:** 2022-08-30

**Authors:** Roopa Rani Samal, Kungreiliu Panmei, P. Lanbiliu, Sarita Kumar

**Affiliations:** Department of Zoology, Acharya Narendra Dev College, University of Delhi, New Delhi, India

**Keywords:** *Aedes aegypti*, acetamiprid, resistance, metabolic detoxification, mutation, target site

## Abstract

Despite the continuous use of chemical interventions, *Aedes*-borne diseases remain on the rise. Neonicotinoids are new, safer, and relatively effective pharmacological interventions against mosquitoes. Neonicotinoids interact with the postsynaptic nicotinic acetylcholine receptors (nAChRs) of the insect central nervous system, but the absence of nAChR polymorphism in resistant phenotypes makes their involvement in neonicotinoid resistance uncertain. Thus, an investigation was carried out to understand the role of metabolic detoxification and target site insensitivity in imparting acetamiprid resistance in *Aedes aegypti* larvae. Studies were conducted on the parent susceptible strain (PS), acetamiprid-larval selected strain for five generations (ACSF-5; 8.83-fold resistance) and 10 generations (ACSF-10; 19.74-fold resistance) of *Ae. aegypti*. The larval selection raised α-esterase and β-esterase activities by 1.32-fold and 1.34-fold, respectively, in ACSF-10 as compared to PS, while the corresponding glutathione-S-transferase and acetylcholinesterase activity increased by 22.5 and 2%. The *ace-1* gene in PS and ACSF-10 showed four mismatches in the 1312—1511 bp region due to mutations in the Y455C codon (tyrosine to cysteine) at the 1367th position (TAC→TGC); I457V codon (isoleucine to valine) at 1372 bp and 1374 bp (ATA→GTG); and R494M codon (arginine to methionine) at 1484 bp (AGG→ATG). The R494M mutation was the novel and dominant type, observed in 70% ACSF-10 population, and has not been reported so far. The studies evidenced the combination of metabolic detoxification and target site mutation in imparting acetamiprid resistance in *Ae. aegypti*.

## Introduction

*Aedes aegypti* L. is a widespread disease vector posing a wide range of health risks, particularly in tropical and subtropical areas owing to favorable climatic conditions. Ever since the emergence of various *Aedes-*borne diseases, dengue has become a major public health concern, with reports of 390 million annual dengue infections and 96 million clinically manifested cases (Dey, 2022). Control of dengue and other *Aedes-*borne diseases is primarily based on *Aedes* management owing to the absence of successful vaccines and effective medications. Though, traditional methods, such as mosquito bed nets and window screens, are frequently used by the masses for avoiding human–mosquito contact, chemical-based control interventions are still the preferred measures due to their instant and effective actions (Liu et al., 2006; Kumar et al., 2009).

Several insecticides of different chemical nature and modes of action have been formulated and used against mosquitoes. Overutilization of these chemicals over several decades, however, has developed varying frequency and intensity of resistance in *Aedes* and other species of mosquitoes. Development of notable resistance to various toxicants, such as organochlorines, organophosphates, carbamates, and pyrethroids, used against *Ae. aegypti* and other mosquito species has been reported from different countries, such as Brazil (Lima et al., 2011), China (Li et al., 2015), Colombia (Fonseca-González et al., 2011), India (Kushwah et al., 2015), Malaysia (Ishak et al., 2015), and Thailand (Yanola et al., 2011).

Currently, neonicotinoids, synthetic derivatives of nicotine, are one of the fastest-growing and investigated insecticides against mosquitoes and are considered relatively safer as compared with conventional insecticides. Neonicotinoids interact with postsynaptic nicotinic acetylcholine receptors (nAChRs) of the insect central nervous system (Li et al., 2012). These bind strongly to a minimum of three nAChR subtypes resulting in a biphasic response causing an initial rise in the frequency of spontaneous discharge leading to nerve propagation blockage and rapid cholinergic transmission imparting toxic effects (Anadón et al., 2020). Acetamiprid, a neonicotinoid, reacts with the postsynaptic receptors of neuronal dendrites, ganglia, and muscle junctions of the central nervous system inducing contact as well as gastrointestinal toxicity (Jian-chu et al., 2002; Kimura-Kuroda et al., 2012; Sanchez-Bayo, 2012). Reports have suggested selective toxicity of acetamiprid against vertebrates due to the weak binding of receptor subtypes, non-accumulation in sediments or aquatic organisms, and comparatively eco-safe characteristics than conventional insecticides (Ambrose, 2003).

The adulticidal activity of different neonicotinoids, acetamiprid (2.3%), thiamethoxam (1.5%), and nitenpyram (2%), has been recorded against *Ae. aegypti* at 367 mg/m^2^ dosage (Darriet and Chandre 2013). Application of imidacloprid, at 84 ng/ml (LC_50_), as an efficient larvicide has been proposed against the local population of *Ae. aegypti*, without any concern for quick resistance development (Paul et al., 2006). Similarly, acetamiprid and thiamethoxam have been found efficient against laboratory strains of *Ae. aegypti* in Grenoble, France, but with relatively lower efficacy against resistant strains (Riaz et al., 2013).

The literature study reveals negligible reports of neonicotinoid resistance in mosquitoes (Mouhamadou et al., 2019). Selection of imidacloprid-susceptible strain of *Ae. aegypti* for eight generations (Imida-R) induced a moderate amount of resistance against imidacloprid (Riaz et al., 2013). Nevertheless, a few studies have reported resistance to neonicotinoids in other insects; *Bemisia tabaci* (Gennadius) to acetamiprid and thiamethoxam (Horowitz et al., 2004), *Musca domestica* to thiamethoxam (Kristensen and Jespersen, 2008), *Frankliniella occidentalis* (Pergande) to imidacloprid and acetamiprid (Gao et al., 2014), and *Leptinotarsa decemlineata* (Say) to imidacloprid (Mota-Sanchez et al., 2006). Nonetheless, like other insects, mosquitoes are also capable of developing gradual resistance to any toxicant due to alterations in biochemical and molecular levels. As a result, it is critical to estimate the possibility of resistance development to the insecticide to be recommended for mosquito management, comprehend the mechanism of resistance, and formulate the strategies to counter resistance.

It is known that metabolic detoxification of insecticide causing resistance in insects is contributed by three major enzyme groups, esterases, glutathione-S-transferases, and monooxygenases. Involvement of DDT-dehydrochlorinase in imparting glutathione-S-transferase resistance was first acknowledged in *M. domestica* (Clark and Shamaan, 1984) and subsequently in *Anopheles* and *Aedes* mosquitoes (Grant and Matsumura., 1988; Prapanthadara et al., 1995). Esterases are known to contribute toward organophosphate, carbamate, and pyrethroid resistance in mosquitoes while the role of monooxygenases has been reported in the metabolism of pyrethroids, organophosphates, and, to a minor extent, carbamates (Casida, 2011; IRAC, 2011; Győri et al., 2017). The development and mechanism of neonicotinoid resistance in mosquitoes have not been studied extensively. It has been suggested that presumably, these mechanisms, individually or in combination, may be the cause of neonicotinoid resistance in *Ae. aegypti* as the absence of nAChR polymorphism in resistant phenotypes makes the involvement of these receptors in neonicotinoid resistance uncertain (Bass et al., 2011; Kasai et al., 2014; Ilias et al., 2015)*.*

The presence of multiple resistance mechanisms in mosquitoes may be an obstacle to the future success of mosquito control programs based on ITNs or indoor residual spraying with insecticides. Since the development of insecticide resistance is a multifaceted and vigorous process, metabolic detoxification and acetylcholinesterase target site insensitivity may play a significant role in imparting acetamiprid resistance in *Ae. aegypti*, because of cross-resistance to other classes of insecticide. Thus, the current study aimed to explore the causative factors involved in the development of acetamiprid resistance in *Ae. aegypti* larvae. Larvae of a laboratory strain of *Ae. aegypti*, selected with acetamiprid for 5 and 10 successive generations, possessing 8.83 and 19.74 levels of resistance, were taken for the study. Levels of metabolic detoxifying enzymes, nonspecific esterases, glutathione-S-transferase, and acetylcholinesterase, were estimated to understand their role. In addition, the involvement of acetylcholinesterase target-site insensitivity (*ace-1* gene) in developing acetamiprid resistance in *Ae. aegypti* was estimated. It is believed that these studies may have huge implications in developing successful *Aedes* control tactics and resistance management strategies.

## Materials and methods

### *Aedes aegypti* stock culture

Larvae and adults of *Ae. aegypti* have been colonized in the rearing unit of Acharya Narendra Dev College, New Delhi, India, since 2009. Rearing conditions have been set at 28 ± 1°C temperature, 80 ± 5% relative humidity, and a 12 h:12 h (light: dark) photo-regime under sterile conditions (Warikoo et al., 2012; Samal and Kumar, 2018).

Adult *Ae. aegypti* are housed in netted cages (45 cm × 40 cm × 40 cm) and are provided nutrition through feeding on the juice of deseeded water-soaked raisins. Female mosquitoes are given blood meals from albino mice (procured from the rearing unit of the University of Delhi) on alternate days for at least an hour. The eggs are gathered on the Whatman filter paper strip linings of a plastic bowl filled with dechlorinated water. Larvae are hatched in a plastic tray (25 cm × 30 cm × 5 cm) filled with 1.5–2.0 L of dechlorinated water. Care has been taken not to make the trays crowded limiting to a total of 200 larvae/tray. Larvae are fed upon an artificial diet of powdered dog biscuits and active yeast (3:1 by weight) (Warikoo et al., 2012). Trays are kept free of any dirt and scum by changing the water every day.

### Chemicals used

The technical grade of acetamiprid (99.9% purity) was procured from M/s Sigma-Aldrich, India. Desired concentrations were prepared in ethanol (eMerck) and stored at 4°C. The chemicals used in the estimation of metabolic detoxifying enzymes and target site insensitivity were procured from eMerck, Qualigen, and Sigma-Aldrich.

### Strains of *Aedes aegypti* selected for the studies

Early fourth instars of the parent strain of *Ae. aegypti* were exposed to acetamiprid and LC_50_ and LC_90_ values were calculated (Samal and Kumar, 2021). The strain was selected at the early fourth instar stage by subjecting to acetamiprid selection pressure at the LC_90_ level, as reported in our previous studies (Samal and Kumar, 2021). The selection was carried out for ten successive generations and the resistance level to acetamiprid was estimated in each generation according to the following equation:

$$\text{Resistance}\;\text{ratio}=\frac{{\text{LC}}_{50}\text{value}\;\text{of}\;\text{acetamiprid}\;\text{against}\;\text{ACSF}\;\text{strain }}{{\text{LC}}_{50}\text{value}\;\text{of }\;\text{acetamiprid}\;\text{against}\;\text{PS}\;\text{strain}}$$ (1)

Following strains, selected for the current study, have been maintained in the laboratory under controlled conditions:

1) Insecticide susceptible strain of *Ae. aegypti* (**PS**) established in the year 2009 without any selection pressure of any insecticide [susceptibility to Acetamiprid: LC_50_ = 0.18799 mg/L; LC_90_ = 1.31547 mg/L]
2) Acetamiprid-selected strain of *Ae. aegypti* (**ACSF-5**) subjected to acetamiprid selection pressure at the larval stage at LC_90_ level for five successive generations and kept under constant selection pressure [susceptibility to acetamiprid: LC_50_ = 1.65916 mg/L; LC_90_ = 4.50887 mg/L; resistance ratio (RR) = 8.83]
3) Acetamiprid-selected strain of *Ae. aegypti* (**ACSF-10**) subjected to acetamiprid selection pressure at the larval stage at LC_90_ level for 10 successive generations and kept under constant selection pressure [susceptibility to acetamiprid: LC_50_ = 3.71057 mg/L; LC_90_ = 10.08811 mg/L; resistance ratio (RR) = 19.74]

### Biochemical characterization of the acetamiprid resistance in *Aedes aegypti*

The development of acetamiprid resistance in *Ae. aegypti* was characterized by estimating levels of various metabolic detoxification enzymes in early fourth instars. Quantification of proteins and detoxifying enzymes; α-esterase, β-esterase, glutathione-S-transferase (GST), and acetylcholinesterase (AChE); was carried out in larvae of PS, ACSF-5, and ACSF-10 strains. The standard WHO protocol to detect insecticide resistance (WHO, 1998) was used with minor modifications (Kona et al., 2018).

Individual larvae of each strain was homogenized in 200 µl of chilled autoclaved water and spun in a refrigerated microfuge at 17,000 × g for 30 s (Hanil science industrial Smart R17 micro-refrigerated centrifuge). Concentrations of proteins, esterases, and glutathione-S-transferase were measured in the supernatant, whilst the AChE level was measured in the crude homogenate. A total of five replicates were carried out, each replicate containing 20 individual larvae (*n* = 100), and each larva was assayed twice.

### Estimation of protein concentration

The 10 µl supernatant of each larval homogenate was taken in a microtiter plate and was added with 300 µl of the BIORAD protein reagent. The plate was incubated for 10 min and scanned at 570 nm with the help of an ELISA plate reader (Tecan i-control, infinite 200pro). A blank was run with water and a standard was run with BSA instead of the larval homogenate.

### Estimation of α-esterase and β-esterase levels

The 10 µl supernatant of each larval homogenate pipetted in the microtiter plate was supplemented with the 200 µl of 3 mM α-naphthyl acetate/β-naphthyl acetate (Sigma Aldrich) and incubated for 15 min. Postincubation, each mixture was added with 50 µl of 6.3 mM fast blue stain (freshly prepared). The visual color changes were interpreted by measuring the absorbance at 570 nm (Brogdon and Dickinson, 1983). A standard was run with respective α-naphthol/β-naphthol (Sigma Aldrich). The esterase activity was expressed as nmol of naphthol/min/mg of protein.

### Estimation of glutathione-S-transferase (GST) levels

The 20µl supernatant of larval homogenate was added with 50 µl of 2 mM GSH and 50 µl of 1 mM of 1-chloro-2,4-nitrobenzene (CDNB) (Sigma Aldrich) in a microtitre plate. Plates were read continuously for 5 min at 340 nm (Brogdon and Barber, 1990). The larval GST activity in *Ae. aegypti* was expressed as nmol/min/mg protein.

### Estimation of acetylcholinesterase (AChE) inhibition

Two replicates of 25 µl of crude insect homogenate taken in a microtiter plate were added with 145 µl of 0.017 M Triton X-100 and 10 µl of 0.01 M dithiobis-2-nitrobenzoic acid (DTNB) (Sigma Aldrich) solution. Subsequently, 25 µl of 0.01 M acetylthiocholine iodide (ASCHI) (Sigma Aldrich) solution was added to one replicate, while 25 µl of 0.01 M ASCHI along with 0.1 M propoxur in a 500:1 ratio was added to the other one. The reaction mixtures were incubated for 1 h and the endpoint reading was taken at 405 nm (Brogdon and Barber, 1987). The endpoint of the reaction was calculated as follows:

$$End\;point=\frac{AChE\;activity\;\left(absorbance\right)\;with\;propoxur}{AChE\;activity\;\left(absorbance\right)\;without\;propoxur}.$$ (2)

Percent inhibition of acetylcholinesterase = 100—(100% × Endpoint).

### Statistical analysis

Data obtained were analyzed using single-way variance analysis (ANOVA) and the means were compared by Tukey’s all pairwise multiple comparison test for statistical significance at *p* < 0.05 using the PAWS (SPSS) software 19.0 program.

### Molecular characterization of the acetamiprid resistance in *Aedes aegypti*

The early fourth instar larvae of *Ae. aegypti* of PS and ACSF-10 strains were evaluated for the possible occurrence of mutational changes in the target protein leading to acetamiprid resistance.

#### Extraction of larval genomic DNA

The genomic DNA was extracted from the early fourth instars using the nucleon spin technique (Angelini et al., 2003). Individual larva of each strain was homogenized in the lysis buffer and mixed with 95:5 of buffer GuEX and Proteinase K solution. The mixture was incubated for 15 min at 37°C and centrifuged at 12,000 × g for 4 min at room temperature. The supernatant was collected and added with the Proteinase K stock solution (10 μl), vortexed, and incubated overnight at 60–65°C. The clear supernatant was subsequently mixed with 400 μl of isopropanol, and put into the nucleoSpin Tissue Column in steps. The mixture was centrifuged at 6,000 × g for 1 min followed by the addition of 500 μl ethanol-containing TE buffer in the spin column and centrifugation at 6,000 × g for 1 min (RT). The mixture was washed twice with buffer and the flow-through was discarded. The washed column was centrifuged for 2 min at 6,000 × g (RT) to completely remove the wash buffer and placed in a 1.5 ml centrifuge tube. The DNA was eluted with 100–200 μl preheated elution buffer (70°C), incubated for 2 min and the mixture was centrifuged at 6,000 × g for 1 min (RT). Finally, the DNA was eluted in 20 μl of elution buffer. The 2.0 μl of DNA was run on a gel to check for isolation, and 2.0 μl was utilized to perform PCR quality checks.

#### Identification and detection of mutational changes in the acetylcholinesterase (ace-1) gene

The complete sequence of the *ace-1* gene, accession no. AJ621915.1 was obtained in the FASTA format from NCBI (https://www.ncbi.nlm.nih.gov/nuccore/AJ621915.1/). The primers with 55–60°C Tm and high GC content were constructed using primer three plus software (Table 1).

**TABLE 1:** Primers used in the study.

Primer	Sequence	No. of nucleotides	GC content	Size of amplicon
Forward primer	CGA​TAA​CGA​ATG​GGG​AAC​G	19	52.63	528 bp
Reverse Primer	TCA​GAG​GCT​CAC​CGA​ACA​CA	19	57.89	

The DNA sample was amplified using RT-PCR. The reaction mixture consisted 1 µl each of the purified genomic DNA, forward primer, reverse primer, and Taq polymerase, added with 3 µl of 10 mm dNTP mixture, 5 µl of 10X assay buffer, and 38 µl of autoclaved water. The constituents were thoroughly mixed in a PCR tube by a short spin at 6000 × g for 20 s followed by loading into a thermocycler (Eppendorf).

#### Purification of the amplified/PCR product

Amplified products were purified by removing unused dNTPs and primers using 5 μl of the PCR product. The ExoSAP-IT™ PCR Product Cleanup Reagent (Thermo Fisher) was used for enzymatic cleanup of PCR amplicon by hydrolysis of excess primers and nucleotides in a single step.

#### Sequence alignment data analysis

The amplified product was sequenced by GeneOmbio Technologies Pvt. Ltd. Baner, Pune, India. BioEdit v7.0.5 was used to edit DNA sequences (Ibis Therapeutics, Carlsbad). Nucleotide and protein blast followed by sequence alignment with Clustal W was used to determine the mutation of the AJ621915.1 *ace-1* gene as well as genotype variation (MEGA five Software).

## Results

Current investigations employed early fourth instar larvae of *Ae. aegypti* reared in the laboratory. Susceptible strains (PS) and strains selected with acetamiprid at LC_90_ level for five (ACSF-5; RR = 8.83) and ten (ACSF-10; RR = 19.74) successive generations at the early fourth instar stage were investigated for biochemical and molecular characterization of acetamiprid resistance.

### Estimation of α-esterase and β-esterase levels

**FIGURE 1:**
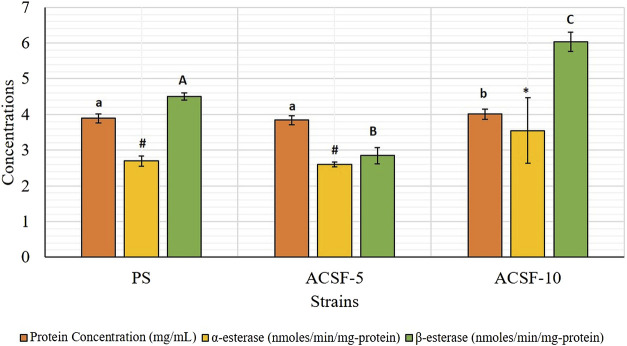
Comparative protein concentration and the esterase activity in PS (parent susceptible), ACSF-5 (acetamiprid larval-selected filial-5), and ACSF-10 (acetamiprid larval-selected filial-10) strains of *Ae. aegypti*. Concentrations of protein/respective esterase indicated by different letters on bars are significantly different (*p* < 0.05); computed by one-way ANOVA followed by Tukey’s all pairwise multiple comparison test.

Corresponding to total protein in the larval body (3.8876 mg/ml), higher mean β-esterase activity (4.5040 nmol/min/mg protein) was recorded in the PS strain as compared to the mean α-esterase activity (2.6943 nmol/min/mg protein) (Table 2). Larval selection with acetamiprid for five generations did not increase the α-esterase activity significantly (*p* > 0.05), but increased significantly (31.6%) after 10 generations of selection (Table 2; Figure 1). In comparison, the level of β-esterases was reduced by 36.82% in ACSF-5 (*p* < 0.05) but elevated by 34.11% (*p* < 0.05) in ACSF-10 (Table 2; Figure 1).

**TABLE 2:** Comparative mean protein concentration and α-esterase activity in parent and acetamiprid larval-selected strains of *Aedes aegypti.*

Strain	Protein concentration (mg/ml) ± SEM	α-esterase (nmol/min/mg protein) ± SEM	β-esterase (nmol/min/mg protein) ± SEM
**PS**	3.8876 ± 0.1327 a	2.6943 ± 0.1471 a	4.5040 ± 0.1002 a
**ACSF-5**	3.8379 ± 0.1281 a	2.6007 ± 0.0615 a	2.8457 ± 0.2223 b
**ACSF-10**	4.0057 ± 0.1403 b	3.5458 ± 0.0917 b	6.0407 ± 0.2639 c

PS, parent susceptible strain of *Ae. aegypti*; ACSF-5, acetamiprid larval-selected filial-5 of *Ae. aegypti*; ACSF-10, acetamiprid larval-selected filial-10 of *Ae. aegypti*. Each strain had five replicates. Each replicate consisted 20 larvae (N = 100 larvae); SEM: standard error of mean; figures in each column followed by different letters are significantly different (*p* < 0.05); one-way ANOVA followed by Tukey’s all pairwise multiple comparison test.

**FIGURE 2:**
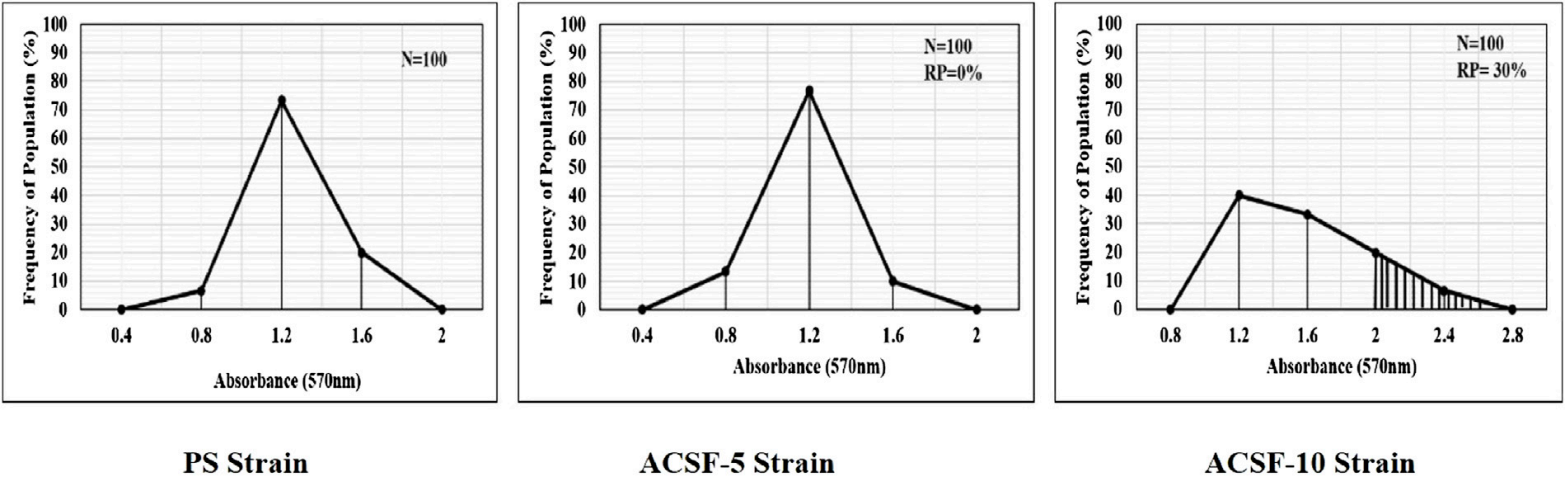
Frequency distributions of absorbance value (570 nm) as the α-esterases activity (nmol/min/mg of protein) in the larvae of PS (parent susceptible strain), ACSF-5 (acetamiprid larval-selected filial-5), and ACSF-10 (acetamiprid larval-selected filial-10) strains of *Aedes aegypti* (*n* = 100). The susceptibility threshold is based on the maximum absorbance in the PS strain. The shaded region represents the resistant population (above the threshold). *n*= number of larvae; RP = resistant population.

**FIGURE 3:**
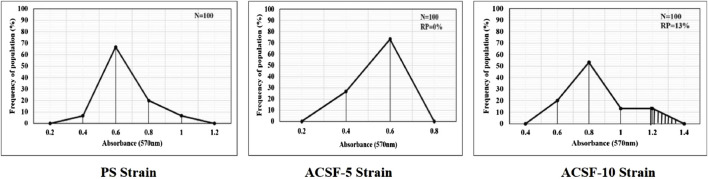
Frequency distributions of absorbance value (570 nm) as the β-esterase activity (nmol/min/mg of protein) in the larvae of PS (parent susceptible strain), ACSF-5 (acetamiprid larval-selected filial-5), and ACSF-10 (acetamiprid larval-selected filial-10) strains of *Aedes aegypti* (*n* = 100). The susceptibility threshold is based on the maximum absorbance in the PS strain. The shaded region represents the resistant population (beyond the threshold). *n*= number of larvae; RP = resistant population.

The frequency distribution profiles of the α-esterase activity were similar in all three strains with a single peak at 1.2 OD, however, 30% of the ACSF-10 population beyond the threshold value possessed elevated α-esterases levels (Figure 2). In comparison, β-esterase profiles of PS and ACSF-5 strains of *Ae. aegypti* had a single peak at OD 0.6, which shifted to OD 0.8 in ACSF-10 with 13% of the ACSF-10 population beyond the threshold value indicating an increased β-esterase activity (Figure 3).

### Estimation of glutathione-S-transferase (GST) levels

**FIGURE 4:**
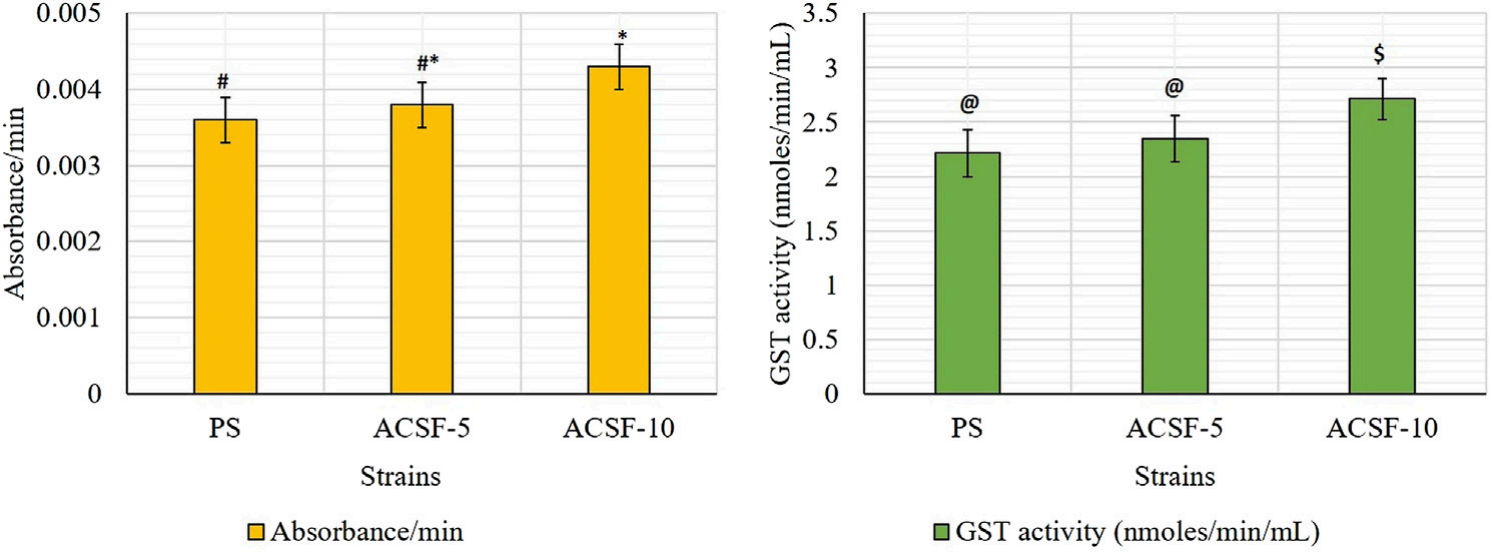
Comparative absorbance/min and the GST activity in PS (parent susceptible), ACSF-5 (acetamiprid larval-selected filial-5), and ACSF-10 (acetamiprid larval-selected filial-10) strains of *Aedes aegypti* (*n* = 100). The absorbance/GST activity indicated by different letters on bars are significantly different (*p* < 0.05); computed by one-way ANOVA followed by Tukey’s all pairwise multiple comparison test.

**FIGURE 5:**
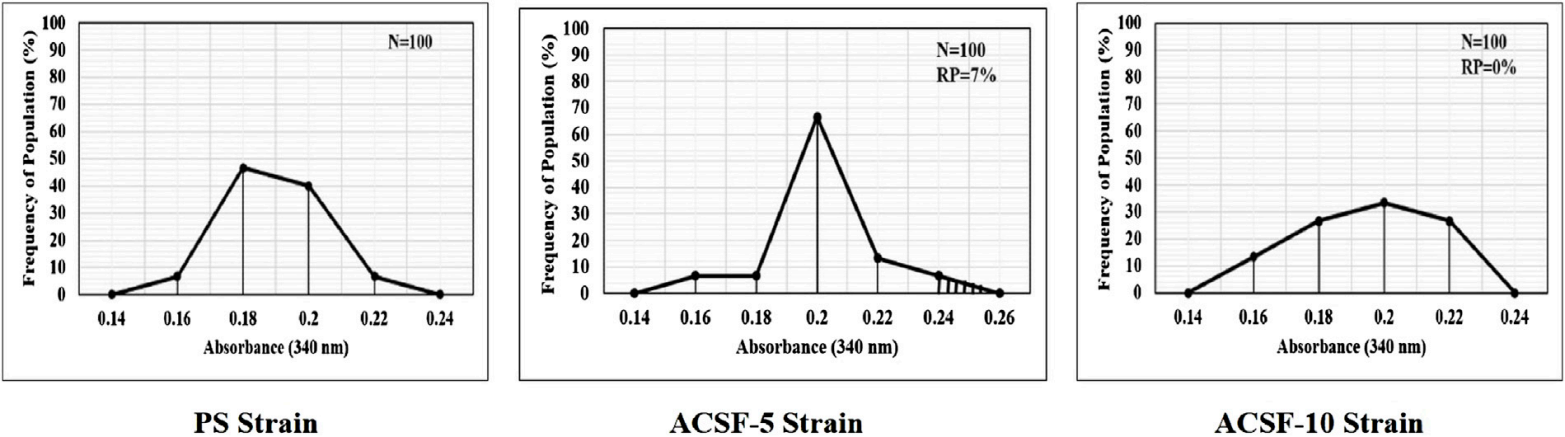
Frequency distribution of absorbance value (340 nm) as the GST activity in the larvae of PS (parent susceptible strain), ACSF-5 (acetamiprid larval-selected filial-5), and ACSF-10 (acetamiprid larval-selected filial-10) strains of *Aedes aegypti* (*n* = 100). The susceptibility threshold is based on the maximum absorbance in the PS strain. The shaded region represents the resistant population (beyond the threshold). *n*= number of larvae; RP = resistant population.

The mean GST kinetics in the PS larvae showed 0.0036 absorbance. The first minute GST activity recorded was 1.8875 nmol/min/ml which gradually increased to 2.7333 nmol/min/ml (*p >* 0.05) after 5 min (Table 4). The selection with acetamiprid did not alter the GST activity initially (2.5208–2.5583) significantly (*p >* 0.05), nonetheless a significant increase (*p <* 0.05) was recorded after 5 min (2.5292–3.0558) with respective 5.94 and 22.49% rise in ACSF-5 and ACSF-10 in comparison to PS (Table 3; Figure 4). The frequency profiles of the GST activity demonstrated insignificant peaks at 0.18 OD and 0.2 OD in PS; a distinct peak at 0.2 OD with 7% larvae beyond the threshold in ACSF-5 and non-prominent, the highest point at 0.2 OD in ACSF-10 (Figure 5).

**TABLE 4:** Mean percent activity and percent inhibition of the AChE activity in parent and acetamiprid-selected larvae of *Aedes aegypti.*

Strain	Endpoint of AChE activity (OD) ± SEM	% Activity of AChE ± SEM	% Inhibition of AChE activity ± SEM
**PS**	0.3801 ± 0.0296 a	38.0122 ± 2.9609 a	61.9878 ± 2.9609 a
**ACSF-5**	0.3819 ± 0.0233 a	38.1890 ± 2.3250 a	61.8110 ± 2.3250 a
**ACSF-10**	0.4093 ± 0.0413 a	40.9308 ± 4.1312 a	59.0692 ± 4.1312 a

PS, parent susceptible strain of *Ae. aegypti*; ACSF-5, acetamiprid larval-selected filial-5 of *Ae. aegypti*; ACSF-10, acetamiprid larval-selected filial-10 of *Ae. aegypti*. Each strain had 5 replicates. Each replicate consisted 20 larvae (N=100 larvae); SEM: standard error of mean; figures in each column followed by different letters are significantly different (p < 0.05); one-way ANOVA followed by Tukey’s all pairwise multiple comparison test.

**TABLE 3:** Mean kinetics of GST in parent and acetamiprid larval-selected strains of *Aedes aegypti.*

Strain	Absorbance/min ± SEM	GST activity (nmol/min/ml) ± SEM
**PS**	0.0036 ± 0.0003 a	2.2158 ± 0.2176 a
**ACSF-5**	0.0038 ± 0.0003 ab	2.3475 ± 0.2114 a
**ACSF-10**	0.0043 ± 0.0003 b	2.7142 ± 0.1881 b

PS, parent susceptible strain of *Ae. aegypti*; ACSF-5, acetamiprid larval-selected filial-5 of *Ae. aegypti*; ACSF-10, acetamiprid larval-selected filial-10 of *Ae. aegypti*. Each strain had 5 replicates. Each replicate consisted 20 larvae (N=100 larvae); SEM: standard error of mean; figures in each column followed by different letters are significantly different (p < 0.05); one-way ANOVA followed by Tukey’s all pairwise multiple comparison test.

### Estimation of acetylcholinesterase (AChE) inhibition

**FIGURE 6:**
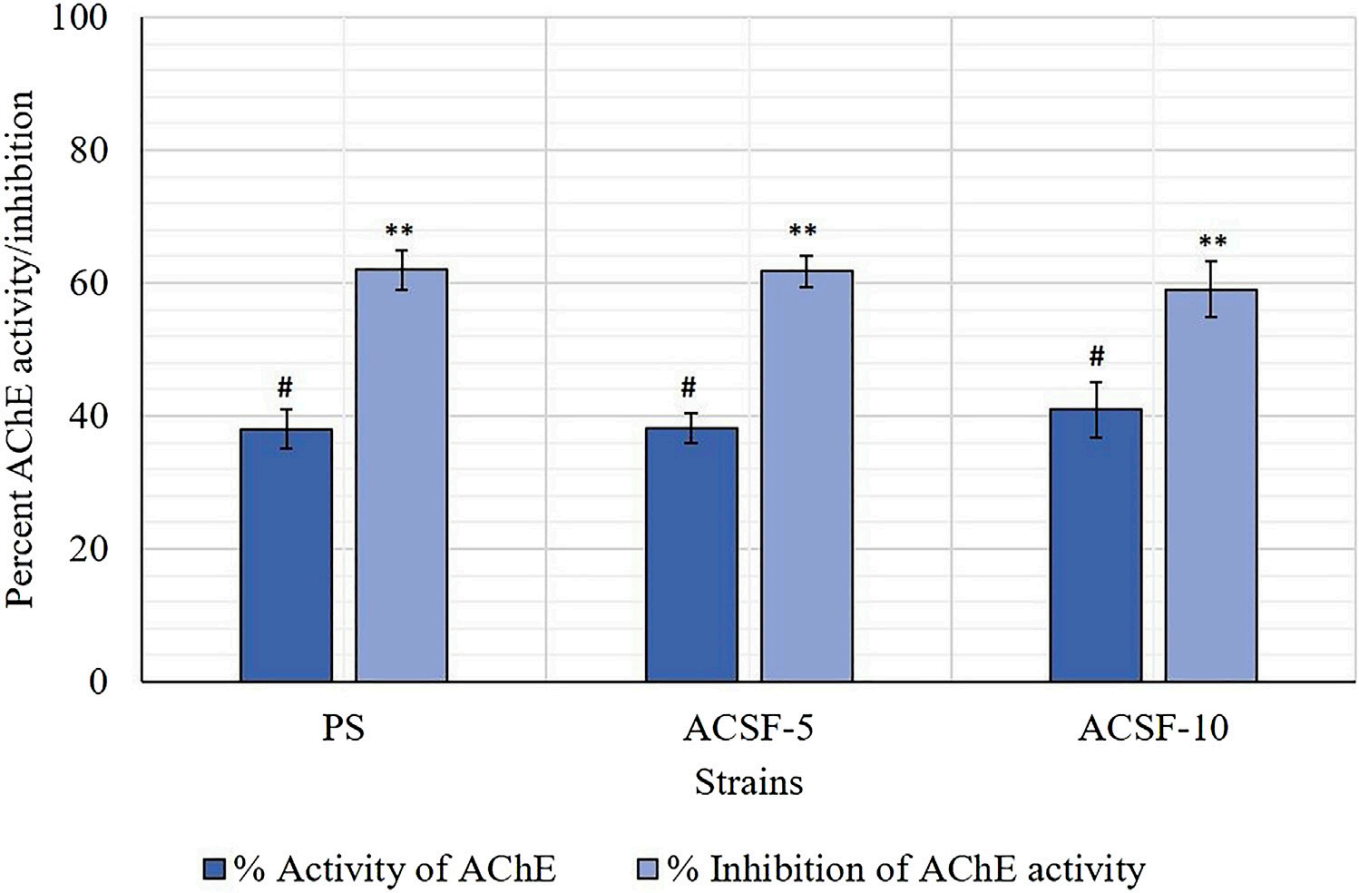
Comparative percent inhibition of the AChE activity in PS (parent susceptible), ACSF-5 (acetamiprid larval-selected filial-5), and ACSF-10 (acetamiprid larval-selected filial-10) strains of *Aedes aegypti* (*n* = 100). The AChE inhibition/activity indicated by the same letter on bars are not significantly different (*p* < 0.05); computed by one-way ANOVA followed by Tukey’s all pairwise multiple comparison test.

**FIGURE 7:**
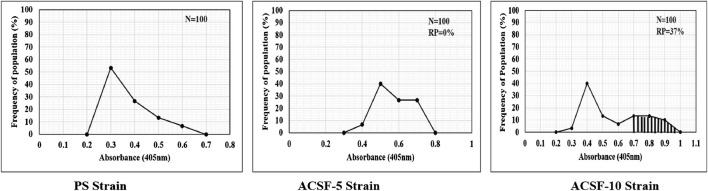
Frequency distribution of the absorbance values of AChE activity inhibition in PS (parent susceptible), ACSF-5 (acetamiprid larval-selected filial-5), and ACSF-10 (acetamiprid larval-selected filial-10) strains of *Aedes aegypti* (*n* = 100). The susceptibility threshold is based on maximum absorbance in PS strain. The shaded region represents the resistant population (beyond the threshold). *n*= number of larvae; RP = resistant population.

The endpoint of the AChE activity in PS, ACSF-5, and ACSF-10 ranged from 0.3801 to 0.4093 OD (*p* < 0.05) (Table 5). The mean %AChE inhibition was in the range of 38% in PS to 41% in ACSF-10 (*p* < 0.05) inducing %AChE activity from 62% in PS to 59% in ACSF-10 (Table 4; Figure 6). The ACSF-5 and ACSF-10 registered 0.18 and 2.92% reduced inhibition of AChE activity, respectively, in comparison to the PS larvae. The maximum population frequency was observed at 0.3 OD in PS and 0.5 OD in ACSF-5. In comparison, ACSF-10 displayed a distinct peak at 0.4 OD with a broad and flat area from 0.6 to 0.8 OD, and 37% of larvae present beyond the susceptible threshold (Figure 7).

**TABLE 5:** Analysis of genotype and phenotype correlation of G1484 T mutation in protein sequence of *Aedes aegypti* based on the mutation frequency in the DNA sequence alignment.

Strain	Sample size	G1484T genotype	
		**GT**	**GG**
		**Phenotype**	
		**^a^** **R**	**^b^** **S**
**PS**	50	0	50
**ACSF-10**	50	35	15
**Frequency (%)**	70%	30%	

a R: resistant phenotype.

b S: susceptible phenotype; PS: parent susceptible strain; ACSF-10: acetamiprid larval selected Filial-10.

### Identification and detection of mutational changes in the acetylcholinesterase (*ace-1*) gene

**FIGURE 8:**
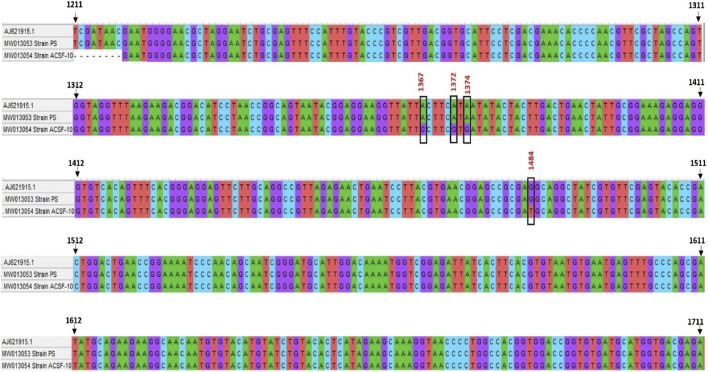
Alignment of nucleotide sequence of *ace-1* gene of PS (parent susceptible) and ACSF-10 (acetamiprid larval-selected filial-10) strains with rock strain of *Aedes aegypti* (accession no. AJ621915.1) using Clustal W (MEGA five software) (*n* = 50).

The amplified genomic DNA of PS and ACSF-10 yielded a PCR amplicon of 528 bp (Table 1). The sequences obtained were submitted to GENBANK with accession numbers MW013053 and MW013054. Nucleotide sequences of the *ace-1* gene of PS and ACSF-10 aligned with the sequence of the *Aedes aegypti* Rock Strain (Accession No. AJ621915.1) is depicted in Figure 8.

**FIGURE 9:**
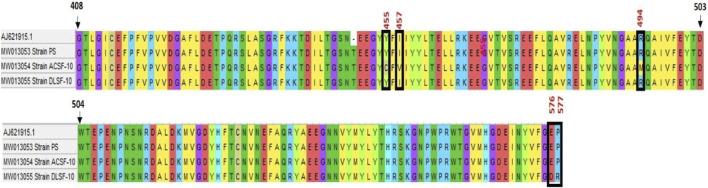
Alignment of translated protein sequence of *ace-1* gene in PS (parent susceptible) and ACSF-10 (acetamiprid larval-selected filial-10) strains with the rock strain of *Aedes aegypti* (accession No.—AJ621915.1) using Clustal W (MEGA five software) (*n* = 50).

The *ace-1* gene sequence comparison of PS and ACSF-10 showed four mismatches in the 1312 bp to 1511 bp region. Three mismatches were due to adenine being replaced by guanine (A → G; A1367G, A1372G, and A1374G), while the fourth was because of the replacement of guanine by thymine (G → T; G1484T) (Figure 8). Posttranslational analysis of open reading frames (ORFs) showed respective mutations in the Y455C codon (Tyrosine to Cysteine) at the 1367th position (TAC → TGC); in the I457V codon (Isoleucine to Valine) at 1372 bp and 1374 bp (ATA → GTG); and in the R494M codon (Arginine to Methionine) at 1484 bp (AGG to ATG) (Figure 9).

Among these mutations, the R494M mutation was observed as a novel and dominant mutation in ACSF-10 present in 70% of the ACSF-10 population, as indicated by the correlation between genotype and phenotype (susceptibility status) of the G1484T codon in *Ae. aegypti* (Table 5).

## Discussion

Vector control strategies, with the introduction of new interventions, go beyond mosquito nets and indoor residual spray taking into account local specificities. Though the application of larvicides is a significant means in mosquito control programs, its implementation involves complicated logistics and efforts leading to the utilization of alternates or novel chemicals. Thus, the present study investigated a neonicotinoid, acetamiprid, for the management of an Indian strain of *Ae. aegypti*, and assessed the mechanism of resistance development in *Ae. aegypti* against acetamiprid in order to devise a strategy to deal with the problem.

Insecticide resistance, considered a preadaptive phenomenon, has emerged as the greatest hindrance to controlling disease vectors. The prolonged and frequent usage of insecticides in crop fields, residential areas, and in public health programs has led to the development of insecticide resistance in mosquitoes by selecting resistant while eliminating susceptible individuals (Uragayala et al., 2015). It is suggested that insects that get selected post-insecticide exposure survive the stress due to altered genome and carry the genetic variance to the successive generation contributing to the resistance gene pool (Faucon et al., 2015). Gradual and sequential selection increase the proportion of resistant organisms which finally outweigh the susceptible population.

Although mechanisms involved in imparting insecticide resistance in vectors are similar across all vector taxa, yet each kind of resistance is unique and comprises a multifaceted resistance foci pattern. Thus, surveillance of vector populations and assessment of their susceptibility to insecticides is significant to design effective control programs (Brogdon and McAllister, 1998). Monitoring insecticide resistance and identifying the underlying mechanism(s) becomes crucial in targeting resistant heterozygotes/homozygotes in the field to manage the mosquito population. The literature though reports the occurrence of increased tolerance of neonicotinoids in the lepidopterans and hemipterans due to their overuse in the fields (Brengues et al., 2003; Ponlawat and Harrington, 2005; Khan and Akram, 2019), yet the resistance, specifically acetamiprid, has not been investigated in mosquitoes. The Arthropod Pesticide Resistance Database (APRD) lists more than 500 cases of resistance to neonicotinoids but not a single report against *Aedes* (Mota-Sanchez and Wise, 2022). Thus, an investigation was conducted to understand acetamiprid resistance development in *Ae. aegypti* which resulted in 19.74-fold resistance after 10 generations of successive selection of the parent strain at the larval stage (Samal et al., 2022).

Resistance to neonicotinoids, like any other insecticide, involves either a target site modification due to a gene polymorphism, or an increase in the insecticide degradation by the action of rising titers of metabolic enzymes (Bass et al., 2011; Kasai et al., 2014; Yang and Liu, 2014). Neonicotinoids are known to target acetylcholine receptors (AChR) in insects, however, their involvement in inducing neonicotinoid resistance remains undefined due to the absence of nAChR polymorphism in resistant phenotypes (Ilias et al., 2015). The metabolism-based mechanisms, involving esterases, CYP450s, and glutathione-S-transferase (GSTs) combined with insensitive AChE have been reported in the development of pyrethroid resistance in mosquitoes (Safi et al., 2017). Thus, the current study investigated the possible role of metabolic detoxification enzymes; ɑ-esterases and β-esterases, glutathione-S-transferase and acetylcholinesterase in the development of acetamiprid resistance in *Ae. aegypti*.

Esterases, a group of heterogeneous enzymes, are present in most organisms. The amplification and/or occasional overexpression of esterase genes increases the production of detoxification proteins negating the effects of toxicants (Vaughan and Hemingway, 1995; Raymond et al., 1998). Continuous selection pressure of acetamiprid increased the ɑ-esterase activity by 1.32-fold and β-esterase by 1.34-fold in ACSF-10 as compared to the susceptible strain suggesting their significant role in acetamiprid hydrolysis and detoxification. Studies implicating the role of esterases in inducing resistance in mosquitoes to different groups of insecticides, primarily pyrethroids and except neonicotinoids, are available. The elevated esterase activity has been reported in deltamethrin-resistant and permethrin-resistant *Ae. aegypti*, in Guerrero State, Mexico (Aponte et al., 2013). However, the insignificant role of esterases in causing pyrethroid resistance has been reported in an Indian strain of *Ae. aegypti* (Sahgal et al., 1994). In contrast, similar results demonstrating elevated esterase levels in *Ae. aegypti* have been reported in north-east Thailand and south-east Asia (Amelia-Yap et al., 2018). Interestingly, in a Santiago de Cuban strain of *Ae. aegypti*, a rise in the esterase frequency from 0.12 to 0.63 was recorded after six generations of deltamethrin selection, which reduced to 0.38 after 12 generations of selection (Rodriguez et al., 2002).

The current study also investigates the role of glutathione-S-transferase in imparting acetamiprid resistance in *Ae. aegypti*. The assay showed a1.22-fold increased GST activity in the ACSF-10 strain and a 1.1-fold increase in the ACSF-5 strain of *Ae. aegypti*. Several studies have shown elevated levels of glutathione S-transferase in insecticide-resistant insects suggesting their role in inducing resistance; most of the studies implicated elevated levels in DDT-resistant mosquitoes (Grant, 1991; Grant and Hammock, 1992; Gunasekaran et al., 2011). The involvement of GST in imparting acetamiprid resistance in *Ae. aegypti* is significant and needs to be investigated further. In addition to esterases and GSTs, the ACSF-10 strain of *Ae. aegypti* showed 2.92% decreased AChE inhibition whereas the ACSF-5 strain had 0.18% decreased AChE inhibition and, thus, increased the AChE activity. Inhibited AChE decreases the sensitivity of insecticides in insects helping in resistance development (Ayad and Georghiou 1975; Hemingway and Georghiou, 1983).

These results suggest differential involvement of esterases, glutathione-S-transferases, and AChE in the development of acetamiprid resistance in *Ae. aegypti* indicating a multifactorial resistance mechanism. The fluctuating detoxifying enzyme level suggests the correlation between selected mechanisms and the metabolic resistance which may be reversed when insecticide pressure ceases. Earlier studies have suggested the role of monooxygenases in the development of acetamiprid resistance in *Ae. aegypti* which can be reversed by the use of Piperonyl butoxide as a synergist (Samal et al., 2022).

Apart from the involvement of detoxifying enzymes in imparting resistance, alteration in the target site is also one of the potential and major mechanisms involved with the resistance. The study, thus, examined the target-site insensitivity for acetylcholinesterase (*ace-1* gene) in the ACSF-10 strain revealing mutations in *ace-I* which is considered a target of majorly the organophosphates. The results showed the occurrence of mutations in the Arg → Met (R494M) codon, Tyr → Cys (Y555C) codon, and Iso → Val (I457V) of the ACSF-10 strain of *Ae. aegypti* which possibly caused the target site insensitivity leading to the development of resistance. Different species of mosquitoes exhibit different mutations linked to the development of insecticide resistance. Higher expression and mutation in the *ace-1* gene encoding the acetylcholinesterase enzyme (AChE1) has imparted OP and carbamate resistance in *An. gambiae* and *Cx. pipiens* (Alout et al., 2008). In contrast, *ace-1* gene mutations associated with an insensitive AChE were not observed in *Ae. aegypti* (Weill et al., 2002).

The report of a single mutation (G119S) associated with insecticide resistance exists in *Cx. pipiens* and *An. gambiae* (Weill et al., 2004). The most common resistance mutation observed in *Cx. pipiens* is G119S (GGC → AGC) located near the catalytic site of the *ace-1* gene imparting high insensitivity to carbamates (Weill et al., 2003) by reducing the AChE1 activity in cholinergic synapses (Alout et al., 2008). The Gly → Ser (G119S) substitution has also been reported in other *Culex* species, *Cx. vishnui* and *Cx. quinquefasciatus*, for organophosphate resistance (Weill et al., 2002; Weill et al., 2003; Liu et al., 2006). In contrast, the G119S mutation was not observed in *An. stephensi* resistant to temephos (Soltani et al., 2015). It has been proposed that possibility of the G119S mutation occurrence in *Ae. aegypti* is low because of two independent mutations (C→ A and C→G) in DNA (Weill et al., 2003). It is also suggested that the maintenance of polymorphic variation in genes may be due to the duplication of *ace-1*. Studies about the maintenance of polymorphism/variation of genotypes in the population have been reported in *Cx. pipiens* (Labbé et al., 2007) and *An. gambiae* (Djogbenou et al., 2009).

These mechanisms, either separately or together, are thought to constitute the root of the development of cross-resistance in ACSF strains of *Ae. aegypti*. Although acetylcholine receptors (nAChR) are known to be a target for neonicotinoids in insects, the role of these receptors in neonicotinoid resistance is unclear because resistant phenotypes lack nAChR polymorphism (Ilias et al., 2015). The role of AChE in conferring acetamiprid resistance in *Ae. aegypti* may be related to the development of cross-resistance to the related class of insecticide as the development of insecticide resistance is a diverse and active process. The current study is the first report on the molecular mechanism of acetamiprid resistance in *Ae. aegypti* based on mutations in *ace-1*. The Arg → Met (R494) mutation observed in the ACSF-10 strain of *Ae. aegypti* was a dominant type and has never been observed earlier. It is proposed that these mutations causing variation in genotypes and translated products supplemented with elevated detoxifying enzymes led to the development of resistance to neonicotinoid. The AChE-insensitive mechanism, together with an overproduced esterase-based mechanism has been found to induce organophosphate and carbamate resistance in a Cuban strain of *Cx. quinquefasciatus* as compared to resistance imparted by individual mechanisms (Bisset et al., 1990).

These findings suggest that acetamiprid resistance in *Ae. aegypti* is a multidimensional and dynamic process that is influenced by a variety of factors. The increased prevalence of resistant *Ae. aegypti* recommends the use of synergists to reverse the resistance or rotation of pesticides with different mechanisms of action to prevent the establishment of resistant homozygotes in the field.

## Conclusion

The rising prevalence of insecticide-resistant *Ae. aegypti* necessitates comprehensive insecticide resistance management for which there is a need to understand the mechanism involved. Current findings assessed the acetamiprid resistance mechanism in *Ae. aegypti* which point to the possible involvement of metabolic detoxifying enzymes supplemented with target site insensitivity (*ace-1*) that caused AGG to ATG mutations at 1484 bp. This study suggests the multifactorial resistance mechanism contributing toward acetamiprid resistance, a complex and dynamic process. These outcomes could aid in understanding and devising mosquito management strategies.

## Data Availability

The datasets presented in this study can be found in online repositories. The names of the repository/repositories and accession number(s) can be found in the article/Supplementary Material.
